# *Azospirillum brasilense* modulates the *Cucumis sativus* root transcriptome under contrasting Fe regimes

**DOI:** 10.3389/fpls.2026.1912672

**Published:** 2026-08-20

**Authors:** Sonia Monterisi, Alessandro Agostini, Fabio Valentinuzzi, Roberto Fattorini, Monica Yorlady Alzate Zuluaga, Stefano Cesco, Youry Pii

**Affiliations:** Faculty of Agricultural, Environmental and Food Sciences, Free University of Bozen/Bolzano, Bolzano, Italy

**Keywords:** *Azospirillum brasilense*, cucumber, Fe deficiency, ferric-chelate reductase activity, PGPR, transcriptomic analysis

## Abstract

Iron (Fe) deficiency limits crop productivity, particularly in alkaline soils where Fe availability is low. Plant growth-promoting rhizobacteria (PGPR), such as *Azospirillum brasilense*, may enhance plant nutrient acquisition. Here, we investigated the transcriptional modulations underlying the interaction between cucumber (*Cucumis sativus*) and *A. brasilense* under Fe sufficient and Fe deficient conditions through physiological and transcriptomic analyses. Bacterial inoculation significantly increased root ferric-chelate reductase activity, particularly under Fe deficiency. Transcriptomic profiling revealed that the bacterium modulated a similar number of genes under both Fe regimes, but with distinct regulatory patterns and only a small set of commonly regulated transcripts, suggesting the existence of a small group of shared transcripts responsive to bacterial inoculation irrespective of Fe status. Gene Ontology enrichment analysis highlighted distinct transcriptional responses in Fe sufficient and Fe deficient plants following *A. brasilense* inoculation. Under Fe sufficiency, *A. brasilense* attenuated stress-related while promoting growth- and metabolism-associated transcripts. Under Fe deficiency, the bacterium induced broader responses involving transcripts related to transport systems, transcriptional regulation, metabolic reorganization, and stress adaptation. In addition, inoculation modulated genes previously associated with Fe mobilization and homeostasis, indicating that the bacterial treatment affects Fe-related processes within this nutritional context. Overall, these findings show that *A. brasilense* stimulates root Fe(III)-reduction capacity and induces distinct transcriptional responses under Fe sufficient and Fe deficient conditions, involving processes beyond canonical Strategy I components and highlighting the importance of plant Fe status in shaping the molecular response to bacterial inoculation.

## 1 Introduction

The continuous growth of the global population and the increasing pressure on agricultural systems highlight the urgent need for sustainable strategies to improve crop productivity and nutrient use efficiency (Țopa et al., 2025). Among minerals, iron (Fe) is an essential micronutrient required for several physiological and biochemical processes in plants, including photosynthesis, respiration, DNA synthesis and nitrogen metabolism (Nikolic et al., 2007; Borlotti et al., 2012). Nevertheless, its availability to plants is frequently constrained in aerobic and alkaline soils, where ferric iron (Fe^3+^) predominates and exhibits low solubility (Robin et al., 2008). Consequently, Fe deficiency represents a widespread agricultural constraint, especially in calcareous soils, significantly affecting crop yield and quality (Hansen et al., 2006; Barker and Stratton, 2015).

In dicot species, such as cucumber (*Cucumis sativus* L.), Fe acquisition relies on Strategy I mechanism, which involves rhizosphere acidification, reduction of Fe^3+^ to Fe^2+^ by ferric-chelate reductase (FRO), and subsequent uptake through specific transporters such as IRT1 (Marschner et al., 2011; Kobayashi and Nishizawa, 2012; Wu et al., 2012). Under severe Fe limitation, however, these responses may not fully satisfy plant Fe requirements. Beneficial rhizosphere bacteria may complement the plant Fe-acquisition machinery by increasing Fe availability in the rhizosphere and by modulating root physiological and molecular responses involved in Fe uptake.

In this context, plant growth-promoting rhizobacteria (PGPR) have emerged as a promising tool to improve plant nutrition and stress tolerance through their interactions with roots and the rhizosphere (Ferreira et al., 2019). Among them, *Azospirillum brasilense* has been widely investigated for its capacity to colonize the rhizosphere and promote plant growth via multiple mechanisms, including biological nitrogen fixation, phytohormone production, nutrient mobilization, and the enhancement of plant tolerance to abiotic stresses (Bashan et al., 2004; Bashan and de-Bashan, 2010; Fukami et al., 2018; Alzate Zuluaga et al., 2022; Zuluaga et al., 2026b). However, the relative contribution of these bacterial traits depends on the plant–microorganism system and the environmental and nutritional conditions. Notably, *A. brasilense* has been shown to enhance Fe availability and uptake through siderophore release, modification of root exudation patterns and root system development, and regulation of genes involved in Fe uptake (Pii et al., 2015, 2016; Marastoni et al., 2019).

Previous studies from our group provided physiological and targeted molecular evidence that the interaction between cucumber plants and *A. brasilense* is influenced by Fe availability. In calcareous soil, bacterial inoculation facilitated the recovery of Fe deficient plants, increasing chlorophyll content, biomass accumulation and leaf Fe concentration, while also altering rhizosphere exudation patterns (Pii et al., 2015). Subsequently, under hydroponic conditions, *A. brasilense* was shown to anticipate or enhance Fe-acquisition-related physiological responses and to increase Fe uptake in both Fe sufficient and Fe deficient plants (Pii et al., 2016). However, targeted expression analyses of canonical Strategy I genes revealed markedly different responses depending on plant Fe nutritional status. In Fe deficient plants, inoculation anticipated and/or enhanced the expression of *CsFRO1*, *CsHA1* and *CsIRT1*, whereas Fe sufficient plants showed distinct or even opposite transcriptional responses, including the lack of *CsFRO1* induction and the downregulation of *CsIRT1* (Pii et al., 2016). These findings suggested that *A. brasilense* may influence Fe acquisition through multiple regulatory mechanisms that depend on the plant nutritional context.

Nevertheless, these previous analyses were restricted to a small set of predefined genes and physiological processes directly associated with Strategy I Fe acquisition. It therefore remained unclear whether the contrasting expression patterns represented the regulation of a few Fe-responsive genes or reflected a broader Fe-status-dependent reprogramming of the root transcriptome. Furthermore, it was not known whether *A. brasilense* induces a conserved transcriptional response irrespective of Fe availability or predominantly activates distinct regulatory programs under Fe sufficient and Fe deficient conditions.

To address this question, we conducted a new and independent experiment reproducing the cucumber–*A. brasilense* biological system and the Fe nutritional conditions previously adopted by Pii et al. (2016). After verifying the expected physiological response through root FCR measurements, we performed genome-wide transcriptomic profiling of roots from inoculated and non-inoculated cucumber plants grown under Fe sufficient and Fe deficient conditions. We hypothesized that the transcriptional response to *A. brasilense* would comprise a limited common component, independent of Fe availability, superimposed on broader regulatory programs shaped by plant Fe nutritional status. Accordingly, the study aimed to identify both the transcripts consistently affected by bacterial inoculation across Fe regimes and the biological processes differentially modulated under Fe sufficiency and deficiency. To isolate the transcriptional response associated with bacterial inoculation, each inoculated treatment was therefore compared with its corresponding non-inoculated control under the same Fe nutritional regime. The study was not intended to provide a comprehensive re-characterization of the basal transcriptional response of cucumber to Fe deficiency. This approach allowed us to extend previous observations beyond classical Strategy I components and to investigate the involvement of nutrient transport, transcriptional regulation, metabolic processes and stress-related responses.

## 2 Materials and methods

### 2.1 Plant material and growth conditions

Cucumber plants (*Cucumis sativus* L.) were grown hydroponically in controlled conditions in a climatic growth chamber with a day/night cycle of 14/10 h and 24 °C/19 °C. The relative humidity was about 70% and the light intensity was 250 μmol m^−2^ s^−1^.

Cucumber seeds were first soaked overnight with 0.5 mM CaSO_4_ and then germinated on a filter paper moistened with 0.5 mM CaSO_4_ solution in darkness for 5 days (Nikolic et al., 2012).

After the germination period, cucumber seedlings were transplanted in pots containing continuously aerated nutrient solution that has been changed every two days. Plants were grown in pots containing 1.5 L of nutrient solution with ten cucumber plants per pot. Each pot was considered as an independent biological replicate. Separate pots were maintained for each treatment and used for the subsequent physiological and transcriptomic analyses. The composition of the hydroponic solution was the following (mM): 2 Ca(NO_3_)_2_, 0.7 K_2_SO_4_, 0.1 KH_2_PO_4_, 0.1 KCl, 0.5 MgSO_4_, and (μM): 10 H_3_BO_3_, 0.5 MnSO_4_, 0.2 CuSO_4_, 0.1 ZnSO_4_, 0.01 (NH_4_)_6_Mo_7_O_24_. The pH of the solution was adjusted to 6 with 1 M KOH. Cucumber plants were grown either in Fe sufficient (Fe was supplied as Fe(III)-EDTA, 80 μM) or in Fe deficient (Fe(III)-EDTA 0 μM) conditions. All plant material, physiological measurements and root samples analysed in the present study were generated specifically in a new and independent experiment. No samples or physiological datasets from Pii et al. (2016) were reused. The experimental conditions were reproduced from that study to provide a comparable physiological framework for the subsequent transcriptomic analysis.

### 2.2 Bacterial growth and plant inoculation

*Azospirillum brasilense* Cd (DSM-1843) was grown in Petri dishes containing solid LB medium (10 g L^-1^ tryptone, 5 g L^-1^ yeast extract, 10 g L^-1^ NaCl and 15g L^-1^ of agar) at 28 °C.

After 3 days of incubation, bacteria were transferred into flasks containing liquid LB medium and grown at a temperature of 28 °C under continuous shaking (180 rpm). After two days of growth, at the onset of the stationary phase, cells were harvested by centrifugation for 15 min at 4500 x g and washed three times with a sterile saline solution (0.85% w/v NaCl). The concentration of the resuspended bacterial pellet has been evaluated spectrophotometrically at 600 nm. Bacterial suspension was used to inoculate the solution, to a final concentration of 10^6^ cfu mL^−1^ of solution (OD_600_ = 0.1). Control sample was treated with the same amount of sterile saline solution. The bacterial suspension was inoculated in the nutrient solution 2 days after the transplanting (DAT) (Pii et al., 2016).

The experimental timeline was as follows: cucumber seedlings were transplanted at day 0, inoculated with *A. brasilense* at 2 DAT, root ferric-chelate reductase activity was assessed at 4 and 6 DAT, and root samples for transcriptomic analyses were collected at 4 DAT (**Supplementary Figure 1**).

### 2.3 Determination of Fe(III) reductase activity

The reduction of Fe(III)-EDTA (FCR activity) by the root system of hydroponically grown cucumber plants was measured colorimetrically using bathophenantroline disulfonate (BPDS) (Vizzotto et al., 1999). The reductase activity was evaluated using five independent biological replicates per treatment (+Fe, -Fe, +Fe + *A. brasilense* and -Fe + *A. brasilense*) at 4 and 6 DAT. For each biological replicate, three plants were randomly selected from the corresponding pot and used for FCR determination. Briefly, roots of intact plants were incubated in the reagent solution (0.5 mM CaSO_4_, 10 mM MES NaOH (pH 5.5), 0.25 mM Fe(III)-EDTA and 0.6 mM BPDS) in the dark at 25 °C. After 30–60 min the absorbance of the substrate solution was recorded at 535 nm. At the end of the assay, roots were blotted dry and their fresh weight was recorded. The amount of Fe(II) formed was calculated from the concentration of the Fe(II)-BPDS_3_ complex using a molar extinction coefficient of 22.1 mM^-1^ cm^-1^. FCR activity was normalized to root fresh weight and the actual incubation time and expressed as nmol Fe(II) g^-1^ root FW h^-1^. FCR activity data were analysed separately at 4 and 6 DAT using two-way ANOVA, with Fe availability (+Fe vs −Fe) and inoculation (non-inoculated vs *A. brasilense*-inoculated) as fixed factors, including their interaction. Tukey’s *post hoc* test was subsequently used for pairwise comparisons among the four treatment combinations. Statistical analyses were performed using R software version 4.0.3.

### 2.4 RNA extraction, cDNA synthesis and transcriptomic analysis

Total RNA was isolated from root of three biological replicates for each treatment at 4 DAT. For each biological replicate, root tissues from all remaining plants within the same pot were pooled prior to RNA extraction. Total RNA was extracted using the Spectrum™ Plant Total RNA kit (Sigma-Aldrich) and quantified by spectrophotometry using NanoDrop™ 1000 (Thermo Scientific). RNA quality was evaluated using Agilent 2100 Bioanalyzer (Agilent). For each sample, the reactions of cRNA synthesis and labelling were carried out using 200 ng of total RNA and the Low Input Quick Amp Labeling Kit, One-Color (Agilent) and Cyanine 3 (Cy3)-CTP fluorescent dye according to the Agilent technical manual (http://www.agilent.com). Cy3-labeled cRNA (1.65 μg) of each sample was hybridized on a custom 4x44K Agilent array according to manufacturer’s manual for 17 h at 65 °C and scanned on Agilent G2565CA Microarray Scanner System (Agilent). Array hybridizations and washing were performed according to manufacturer’s manual (One-Color Microarray-Based Gene Expression Analysis—Low Input Quick Amp Labeling—Protocol). Each subarray allows analysing the expression of 30364 transcripts predicted from the *Cucumis sativus* L. cv. Gy14 (http://cucurbitgenomics.org/organism/7). Probe design was performed using Agilent eArray (http://www.genomics.agilent.com). A complete description of the chip is available at the Gene Expression Omnibus (http://www.ncbi.nlm.nih.gov/geo) under the series entry (GPL34058). The hybridization data of all the samples have been normalized using the value of the 75^th^ percentile and log_2_ transformed. Differentially expressed transcripts were identified using Student’s t-test implemented in MeV software (Saeed et al., 2023). *P*-values were adjusted for multiple testing, and transcripts with an adjusted *p*-value < 0.05 were considered significantly differentially expressed. The primary differential-expression comparisons were defined to assess the transcriptional effect associated with *A. brasilense* inoculation within each Fe nutritional condition: +Fe + *A. brasilense* vs +Fe and −Fe + *A. brasilense* vs −Fe. Because each inoculated treatment was compared with its corresponding non-inoculated control under the same Fe regime, the resulting transcriptional changes represent the response associated with bacterial inoculation within each nutritional context. The microarray data underlying this article are available in the Gene Expression Omnibus (GEO) Database at https://www.ncbi.nlm.nih.gov/geo/, and can be accessed with GSE331093 accession number.

Cucumber transcripts have been annotated through Phytozome *Cucumis sativus* v1.0 annotation. A Principal Component Analysis (PCA) was performed using normalized expression values. The analysis was conducted on the 2,000 transcripts showing the highest variance across all samples. Gene Ontology (GO) enrichment analysis of differentially expressed genes (DEG) was carried out by exploiting agriGO (v2.0) tool (Tian et al., 2017). The DEG has been assigned to GO categories using a FDR (False Discovery Rate)-adjusted *p-*value < 0.05.

### 2.5 Quantitative reverse transcription PCR analysis

Each RNA sample was treated with DNase I treatment using 1000 ng of total RNA and the RQ1 RNase-Free DNase (Promega, Madison, WI, USA) according to the manufacturer’s protocol. Complementary DNA (cDNA) synthesis was performed with the ImProm-II™ Reverse Transcription System (Promega, Madison, WI, USA) in accordance with the supplied instructions. Gene-specific primers, including one housekeeping gene (*Cucsa.193990.1*), were designed and reported in the **Supplementary Table 1**. Quantitative real-time PCR (RT-qPCR) was conducted in triplicate under previously established cycling conditions (Alzate Zuluaga et al., 2022). Relative gene expression levels were determined using the 2^–ΔΔCt^ method, as described by Livak and Schmittgen (2001).

## 3 Results

### 3.1 Plants Fe(III) reductase activity to assess plant physiological status

**Figure 1:**
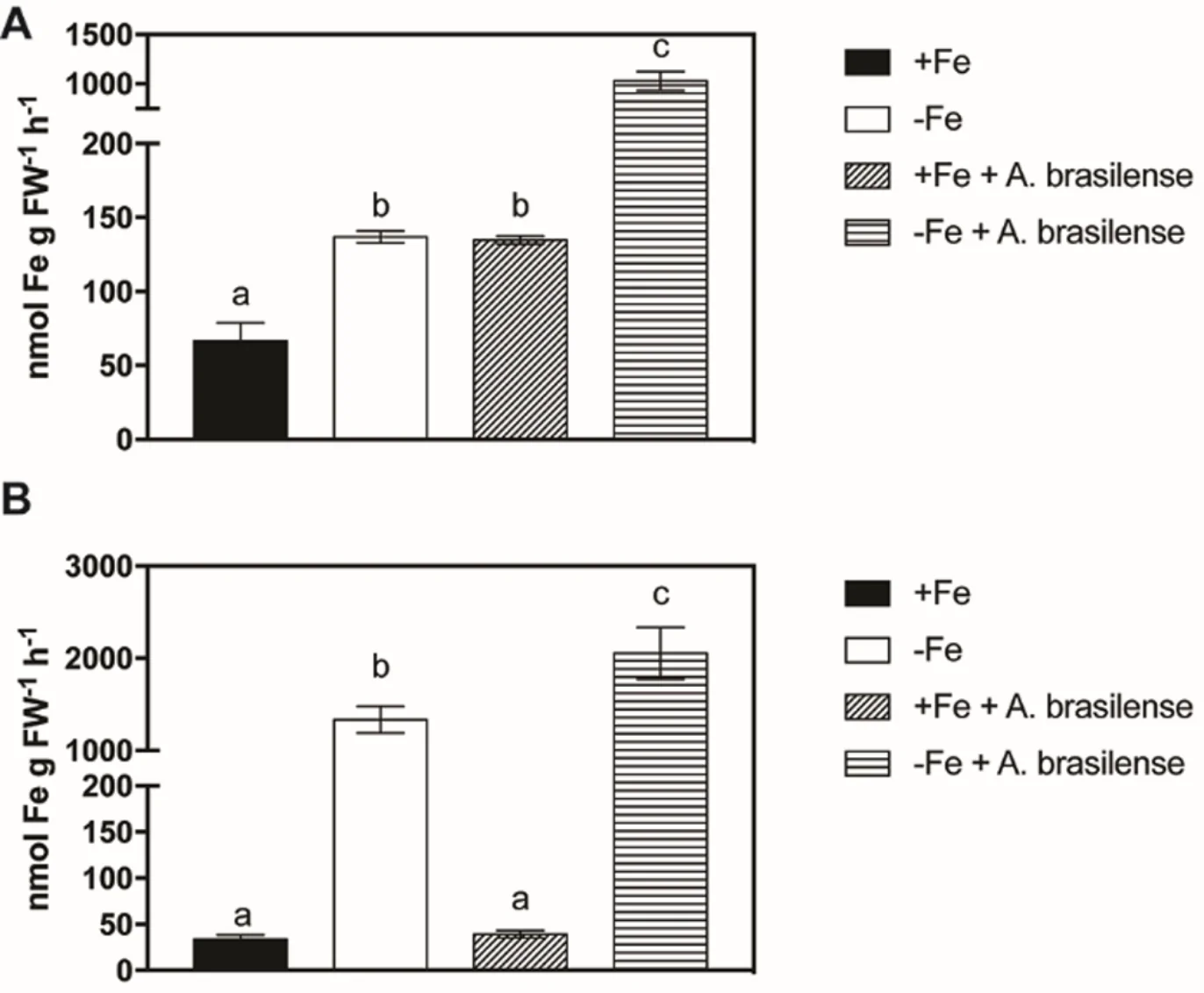
Quantification of Fe(III) reductase activity. **(A)** Fe(III)-EDTA reduction activity in both Fe sufficient and Fe deficient plants, either non-inoculated or inoculated with *A. brasilense*, at 4 DAT. **(B)** Fe(III)-EDTA reduction activity in both Fe sufficient and Fe deficient plants, either non-inoculated or inoculated with *A. brasilense*, at 6 DAT. For each sampling time, the effects of Fe availability, *A. brasilense* inoculation and their interaction were assessed using two-way ANOVA. Tukey’s *post hoc* test was used for pairwise comparisons among the four treatment combinations; different letters indicate significant differences (*p-*value < 0.05). At 4 DAT: Fe availability, *p-*value = 2.40 × 10^-5^; inoculation, *p-*value = 4.36 × 10^-5^; Fe availability × inoculation, *p-*value = 2.15 × 10^-4^. At 6 DAT: Fe availability, *p-*value = 2.05 × 10^-6^; inoculation, *p-*value = 0.0940; Fe availability × inoculation, *p-*value = 0.0636.

To obtain a deeper insight in the interaction between cucumber plants and *A. brasilense*, at 2 DAT both Fe sufficient and Fe deficient plants were inoculated with *A. brasilense* and the physiological response, *i.e.*, activation of the Fe reductase enzymatic (FCR) activity was monitored at 4 and 6 DAT (**Figure 1**).

At 4 DAT, Fe deficient cucumber plants showed a significantly higher FCR activity as compared to Fe sufficient plants (**Figure 1A**). Nonetheless, the inoculation with *A. brasilense* caused a general increase in the FCR activity in both Fe sufficient and Fe deficient plants (**Figure 1A**). In particular, inoculated Fe sufficient cucumber plants displayed a FCR activity comparable to that observed in Fe deficient plants, whilst inoculated Fe deficient plants showed a 10-fold increased FCR activity with respect to non-inoculated Fe starved plants (**Figure 1A**). Two-way ANOVA revealed significant effects of Fe availability (*p*-value = 2.40 × 10^-5^), *A. brasilense* inoculation (*p*-value = 4.36 × 10^-5^) and their interaction (*p*-value = 2.15 × 10^-4^).

At 6 DAT, the Fe deficient plants showed a strong up-regulation in the FCR activity, being about 40 times higher as compared to that detected in the Fe sufficient plants (**Figure 1B**). The inoculation with *A. brasilense* caused in Fe deficient plants a further increase in the FCR activity by about 60% with respect to non-inoculated plants (**Figure 1B**). On the other hand, the treatment with *Azospirillum* at 6 DAT did not increase the FCR activity in Fe sufficient plants (**Figure 1B**). At 6 DAT, two-way ANOVA detected a significant effect of Fe availability (*p*-value = 2.05 × 10^-6^), whereas neither the inoculation effect (*p*-value = 0.0940) nor the Fe availability × inoculation interaction (*p*-value = 0.0636) was statistically significant.

### 3.2 Transcriptomic analysis

Based on the physiological assessment, a transcriptomic analysis was carried out on the Fe sufficient and Fe deficient cucumber plants, both non-inoculated and inoculated with *A. brasilense* at 4 DAT (**Figure 1A**). To obtain a global overview of the transcriptomic dataset, a PCA was performed using the 2,000 transcripts showing the highest variance across all samples (**Supplementary Figure 2**). The PCA showed a clear separation among the four experimental groups, while biological replicates clustered closely together, supporting the robustness of the dataset. The first two principal components explained 35.4% and 17.3% of the total variance, respectively. Notably, inoculated plants exhibited distinct transcriptional profiles from their corresponding non-inoculated controls under both Fe sufficient and Fe deficient conditions, showing that bacterial inoculation was associated with detectable transcriptomic changes within each Fe nutritional regime.

The comparison among treatments aimed at identifying the influence of the inoculation of *A. brasilens*e on the transcriptome of both Fe sufficient and Fe deficient plants. Indeed, *A. brasilense* induced differential expression of 393 transcripts in Fe sufficient plants, of which 213 were up-regulated and 180 down-regulated (**Table 1**). Similarly, in the case of Fe deficient plants, the presence of the PGPR caused the differential modulation of 406 transcripts, 224 of which were up-regulated, whereas 182 down-regulated (**Table 1**).

**Table 1:** Number of differentially expressed transcripts (DET).

Comparison	DET		
	Up-regulated	Down-regulated	Total
+Fe + *A. brasilense* vs +Fe	213	180	393
-Fe + *A. brasilense* vs -Fe	224	182	406

Differentially expressed transcripts have been identified through Student’s t-test and filtered on the basis of an adjusted *p*-value < 0.05.

**Figure 2:**
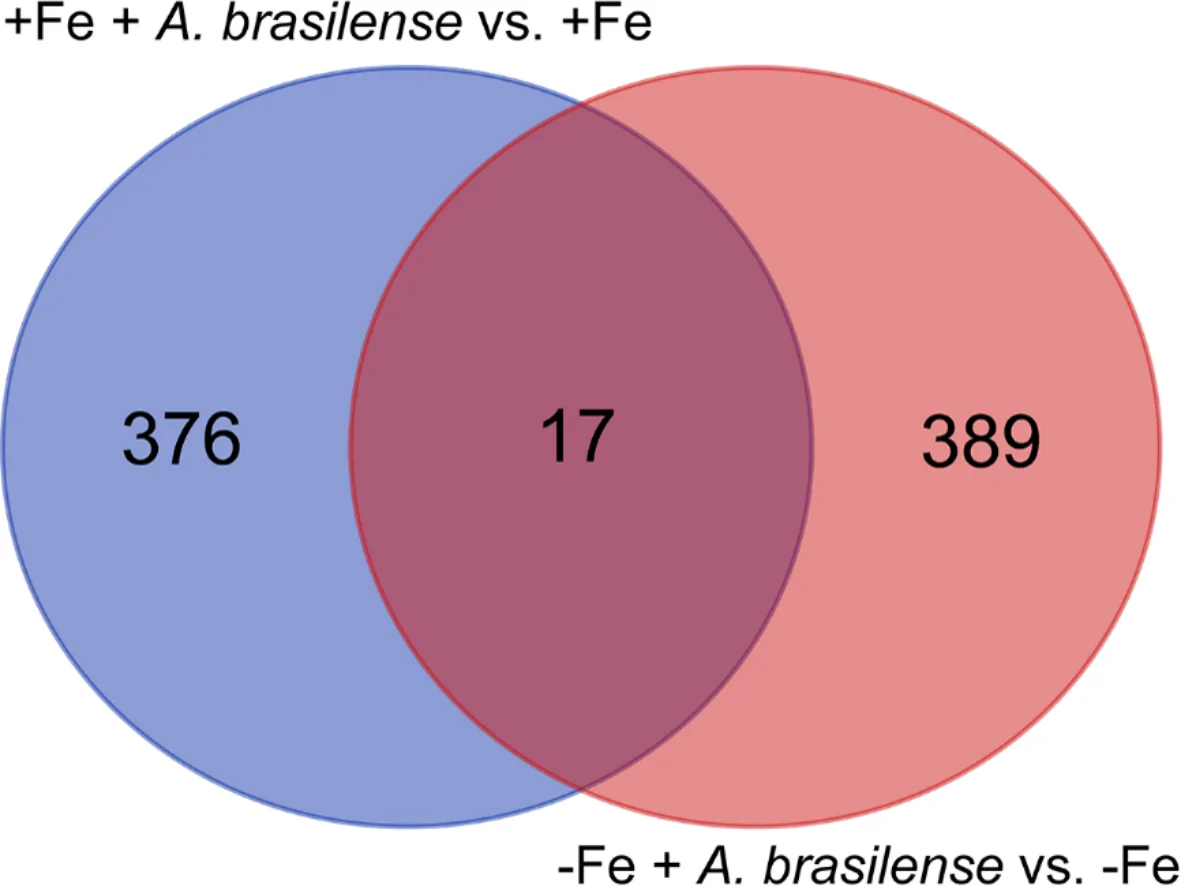
Venn diagram of DET. Uniquely and co-modulated DET between the comparisons +Fe + *A. brasilense* vs +Fe and -Fe + *A. brasilense* vs -Fe.

Comparison of the total number of transcripts modulated by *A. brasilense* inoculation under Fe sufficient and Fe deficient conditions, each normalized to the corresponding uninoculated treatment, revealed that 376 and 389 transcripts were uniquely modulated under Fe sufficient and Fe deficient conditions, respectively (**Figure 2**). Only 17 transcripts were co-modulated in the two comparisons, representing a limited shared response to *A. brasilense* inoculation detected under both Fe nutritional regimes (**Figure 2**). To validate the transcriptomic results, the differential expression of four of these co-modulated transcripts was further assessed by RT-qPCR (**Supplementary Table 2**).

Among the commonly modulated transcripts few overcame the threshold of |log_2_FC|≥ 1 in both the comparisons (**Table 2**). In details, five DET resulted up-regulated, two corresponding to remorin family proteins (*Cucsa.258280.1* and *Cucsa.258280.3*), one to a zinc finger protein jackdaw (*Cucsa.306990.1*), one to a SKU5-like protein (*Cucsa.388290.1*) and the last one to a vanadium-binding protein 2 superfamily (*Cucsa.002310.1*) (**Table 2**). Only one transcript was down-regulated in both the comparisons and it encoded for an AP endonuclease 2 (APEX2) (*Cucsa.043590.2*) (**Table 2**).

**Table 2:** DETs commonly modulated by the comparisons +Fe + *A. brasilense* vs +Fe and -Fe + *A. brasilense* vs -Fe.

DETs co-modulated in +Fe + *A. brasilense* vs +Fe and -Fe + *A. brasilense* vs -Fe			
Transcript ID	log_2_(Fold Change) +Fe + *A. brasilense* vs +Fe	log_2_(Fold Change) -Fe + *A. brasilense* vs -Fe	Description
Cucsa.258280.1	1.92	1.69	Remorin family protein
Cucsa.258280.3	1.88	1.64	Remorin family protein
Cucsa.254640.1	1.67	0.92	Early light-induced protein 1
Cucsa.306990.1	1.56	1.36	Zinc finger protein jackdaw
Cucsa.388290.1	1.41	1.23	protein SKU5-like 3
Cucsa.002310.1	1.18	1.25	Vanadium-binding protein 2 superfamily
Cucsa.254710.1	1.09	0.87	ATP sulfurylase 1
Cucsa.383130.1	0.97	0.78	Multiple Inositol polyphosphate phosphatase 1
Cucsa.083080.1	0.79	1.31	Glucomannan 4-β-mannosyltransferase 9
Cucsa.127090.1	0.78	0.56	Mitogen-activated protein kinase 13
Cucsa.303500.1	0.51	1.56	Copper transport protein family
Cucsa.084230.2	0.39	-0.81	Heat stress transcription factor A-7A
Cucsa.160510.1	-0.38	-0.46	Fmp27 protein domain
Cucsa.162820.1	-0.58	-1.07	WRKY family transcription factor-related
Cucsa.085280.3	-0.68	-0.58	Peptidyl-prolyl cis-trans isomerase H
Cucsa.174360.1	-0.81	0.88	Methylesterase 13
Cucsa.043590.2	-1.86	-1.85	AP endonuclease 2 (APEX2)

The reported transcripts represent all significantly co-modulated transcripts shared between the two comparisons (adjusted *p*-value < 0.05).

### 3.3 Gene ontology functional enrichment

To identify the main functional responses associated with *A. brasilense* inoculation under the two Fe nutritional regimes, differentially expressed transcripts were aggregated at the gene level and subjected to Gene Ontology enrichment analysis for Biological Process (BP) and Molecular Function (MF) categories. Overall, the two nutritional contexts showed distinct functional profiles. Under Fe sufficiency, enriched terms were mainly related to cellular and metabolic processes, RNA biosynthesis and transcriptional regulation, post-translational protein modification, and catalytic and binding functions, with several stress-associated genes being down-regulated. Under Fe deficiency, a broader range of terms was enriched, particularly those associated with membrane transport and localization, transcriptional and biosynthetic regulation, metabolism, and responses to stimulus and stress. To illustrate these patterns without duplicating the complete gene lists reported in **Supplementary Tables 3** and **S4**, only representative genes with |log2FC| ≥ 1 are highlighted below.

#### 3.3.1 *Azospirillum brasilense* inoculated in Fe sufficient cucumber plants

**Figure 3:**
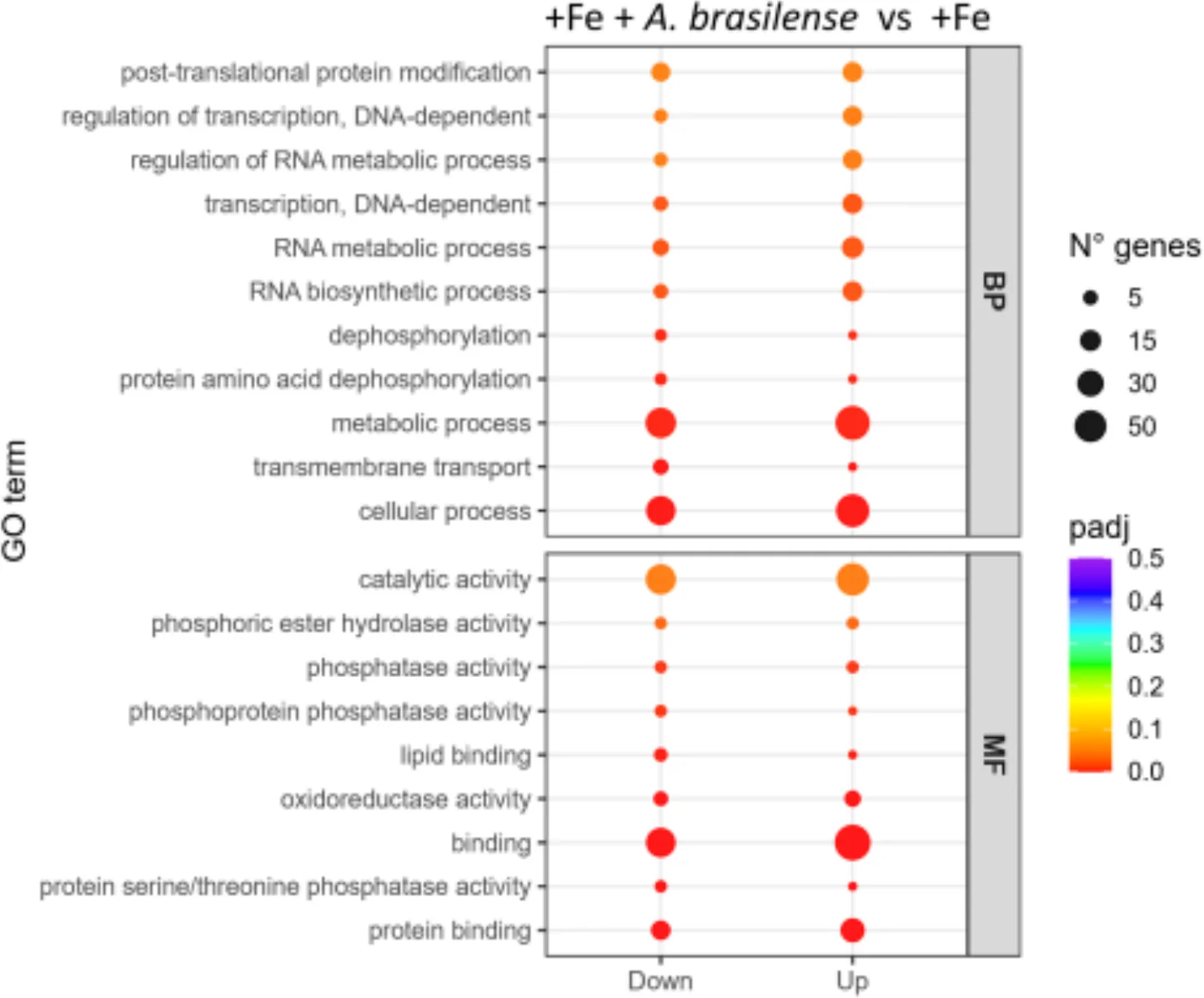
Gene Ontology (GO) enrichment analyses for the +Fe + *A. brasilense* vs +Fe comparison. Displayed GO (adjusted *p-*value < 0.05) belong to the Biological Process (BP) and Molecular Function (MF) categories. For each GO term, ‘Down’ indicate genes with log_2_FC < 0 and ‘Up’ genes with log_2_FC > 0. ‘N° genes’ represents the number up- or down-regulated genes associated with each GO term.

Under Fe sufficient conditions, BP enrichment was mainly associated with *cellular* and *metabolic processes*, which included comparable numbers of up- and down-regulated genes (**Figure 3**). Representative down-regulated genes encoded proteins associated with stress-related signalling and metabolic processes, including an α,α-trehalose-phosphate synthase (*Cucsa.285920*), an ACC synthase (*Cucsa.103460*), a cysteine-rich receptor-like protein kinase (*Cucsa.045540*), an AP endonuclease 2 (*APEX2*; *Cucsa.043590*) and a Fe(II)/2-oxoglutarate dioxygenase (*Cucsa.125280*). In contrast, up-regulated genes included two NADH-dependent nitrate reductases (*Cucsa.026240* and *Cucsa.274720*), two ethylene-responsive transcription factors (*Cucsa.088640* and *Cucsa.129630*), a DOF zinc finger protein (*Cucsa.349890*) and a SKU5-like protein (*Cucsa.388290*) (**Supplementary Table 3**).

Several overlapping BP terms associated with RNA biosynthesis, RNA metabolism and transcriptional regulation were also significantly enriched (**Figure 3**). These terms were mainly supported by the up-regulation of genes encoding a transcription initiation factor TFIID, two ethylene-responsive transcription factors and a DOF zinc finger protein, together with the down-regulation of a bZIP transcription factor. Post-translational protein modification was also enriched and included an up-regulated protein kinase and a down-regulated cysteine-rich receptor-like protein kinase (**Supplementary Table 3**).

Within the MF category, the principal enriched terms were oxidoreductase activity, binding, protein binding and catalytic activity (**Figure 3**). Most of the genes contributing to these terms were also included in the BP categories described above, including the two up-regulated nitrate reductases and the SKU5-like protein, as well as the down-regulated berberine bridge enzyme-like protein and Fe(II)/2-oxoglutarate dioxygenase. The complete list of genes associated with each enriched term is reported in **Supplementary Table 3**.

#### 3.3.2 *Azospirillum brasilense* inoculated in Fe deficient cucumber plants

**Figure 4:**
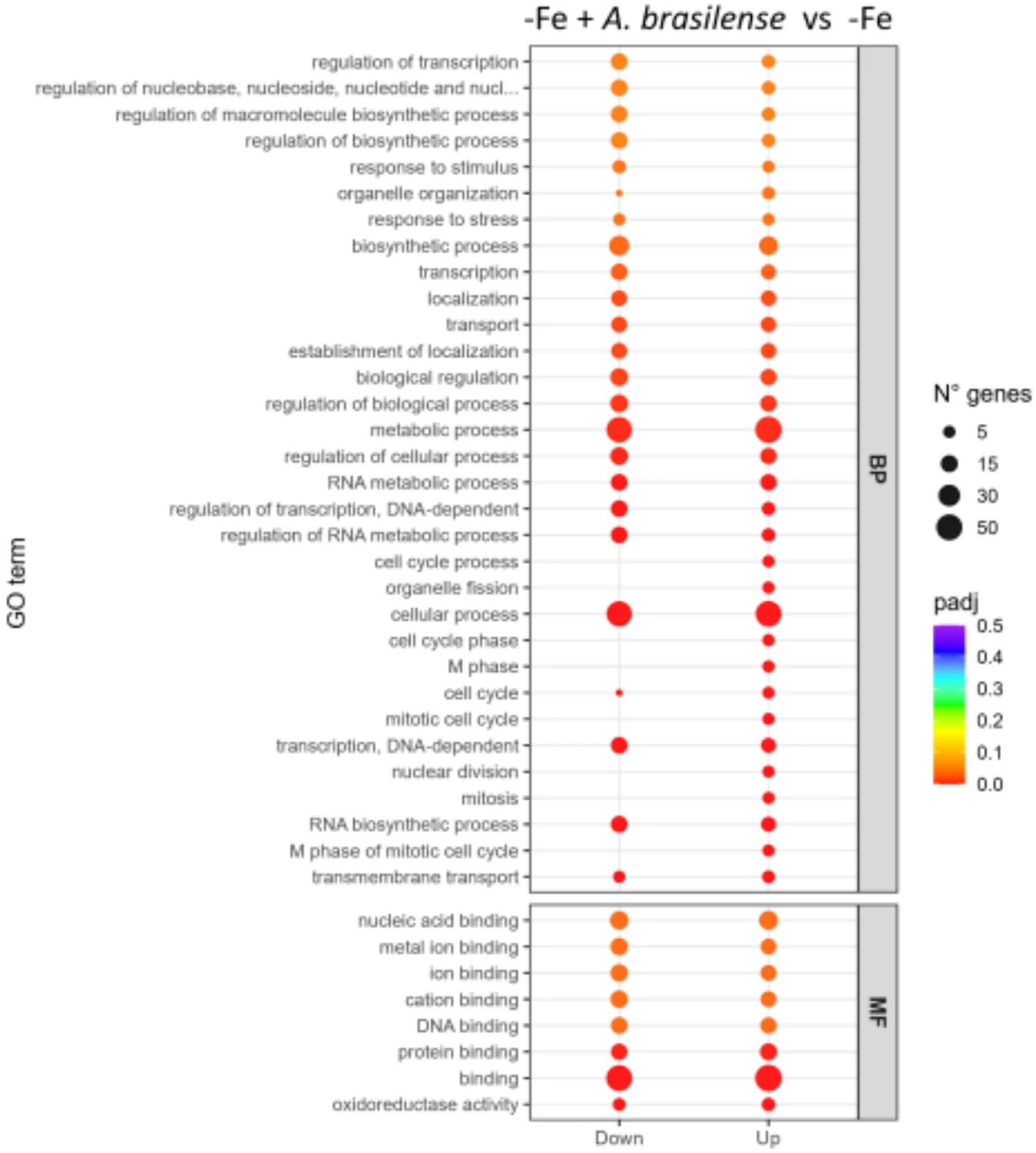
Gene Ontology (GO) enrichment analyses for the -Fe + *A. brasilense* vs -Fe comparison. Displayed GO (adjusted *p-*value < 0.05) belong to the Biological Process (BP) and Molecular Function (MF) categories. For each GO term, ‘Down’ indicate genes with log_2_FC < 0 and ‘Up’ genes with log_2_FC > 0. ‘N° genes’ represents the number up- or down-regulated genes associated with each GO term.

Under Fe deficient conditions, *A. brasilense* inoculation resulted in the enrichment of a broader range of BP terms than under Fe sufficiency (**Figure 4**). Transport-related processes were particularly prominent. Genes encoding a cationic amino acid transporter (*Cucsa.134590*), a sulphate transporter (*Cucsa.029460*), a sugar transporter (*Cucsa.094200*), two copper transport proteins (*Cucsa.303490* and *Cucsa.303500*) and a peptide transporter (*Cucsa.255640*) were up-regulated. In contrast, genes encoding an aquaporin PIP (*Cucsa.124360*) and an equilibrative nucleoside transporter (*Cucsa.133230*) were down-regulated (**Supplementary Table 4**).

Numerous overlapping terms related to RNA biosynthesis, transcription and the regulation of biosynthetic and metabolic processes were also enriched. Representative up-regulated genes included those encoding for AP2/ERF (*Cucsa.150110*), NAC-domain transcription factors (*Cucsa.338700*) and a transcription initiation factor TFIID (*Cucsa.163030*). Conversely, genes encoding a WRKY-related transcription factor (*Cucsa.162820*), two bZIP protein (*Cucsa.234250* and *Cucsa.289120*) and an ERF (*Cucsa.055920*) were down-regulated. These transcription factors contributed to several related regulatory GO terms, indicating extensive modulation of transcriptional regulation under Fe deficiency (**Supplementary Table 4**).

Enriched *cellular*, *metabolic* and *biosynthetic processes* included genes associated with C and N metabolism, Fe homeostasis and cellular remodelling. Among the representative down-regulated genes were those encoding NADH-dependent glutamate synthase (*Cucsa.040650*), a riboflavin synthase (*Cucsa.119030*), a nicotianamine synthase (*Cucsa.092390*), a 6,7-dimethyl-8-ribityllumazine synthase (*Cucsa.327700*), and a Guanosine monophosphate (GMP) synthase (*Cucsa.303210*). Up-regulated genes included those encoding a cellulose synthase-like protein (*Cucsa.309280*), glutamate decarboxylase (*Cucsa.026460*), sterol 3β-glucosyltransferase (*Cucsa.078450*) and two cytochrome P450 proteins (*Cucsa.284370* and *Cucsa.149540*) (**Supplementary Table 4**).

The BP terms *response to stimulus* and *response to stress* were also significantly enriched. These included the up-regulation of two calmodulin-binding protein genes (*Cucsa.090850* and *Cucsa.051190*) and the down-regulation of genes encoding an N-glycosylase/DNA lyase (*Cucsa.060560*) and *APEX2* (*Cucsa.043590*). Within the MF category, enriched terms included *oxidoreductase activity* and several *binding-related functions*, including protein, metal ion, DNA and nucleic acid binding (**Figure 4**). Since the genes contributing to these MF terms largely overlapped with those already included in the BP categories, their complete distribution among the enriched terms is reported in **Supplementary Table 4**.

## 4 Discussion

Plant iron (Fe) deficiency is one of the most widespread nutritional disorders in alkaline and aerobic soils, particularly in calcareous soils, due to the poor solubility of Fe(III) under these conditions. Plant growth-promoting rhizobacteria (PGPR) can contribute to plant Fe acquisition by mobilizing poorly available Fe forms in the rhizosphere and by modulating root Fe-acquisition responses (Parray et al., 2016; Bargaz et al., 2018; Crecchio et al., 2018). Among PGPR, *Azospirillum brasilense* has previously been shown to promote the adaptation of cucumber plants (*Cucumis sativus* L.) to Fe deficiency (Pii et al., 2016). In the present study, we investigated whether the molecular responses induced by *A. brasilense* at the whole-transcriptome level differ according to the Fe nutritional status of the plant.

The preliminary analysis, aimed at assessing plant physiological status through FCR activity, confirmed that, at 4 DAT, Fe deficient plants exhibited higher enzymatic activity compared with Fe sufficient plants, as previously reported (Pii et al., 2016). Notably, *A. brasilense* inoculation increased FCR activity regardless of the cucumber nutritional status, a phenomenon that has also been observed in other plant species inoculated with PGPR (Marastoni et al., 2019; Rahimi et al., 2020; Zhu et al., 2025).

This effect was less pronounced when FCR activity was evaluated at 6 DAT. At this time point, *A. brasilense* inoculation still enhanced enzymatic activity under Fe deficient conditions, whereas no increase was observed in the Fe sufficient ones (**Figure 1B**). This observation is consistent with the findings by Pii et al. (2016), who reported that in Fe sufficient plants *A. brasilense* inoculation induced just a transient increase in FCR activity that subsequently decreased. These results not only confirm our previous observations but also fit into a more complex and partly controversial picture of Fe-acquisition gene regulation by *A. brasilense*, which depends on the plant Fe nutritional status. In Fe-deficient plants, the inoculation with the PGPR anticipates and reinforces the induction of canonical Strategy I components, such as *CsFRO1* and *CsHA1*, whereas in Fe-sufficient plants the transient FCR activation occurs in the absence of *CsFRO1* up-regulation, and even with its down-regulation, implying the involvement of alternative reductases or regulatory routes (Pii et al., 2016). This Fe-status-dependent modulation is further supported by the characterization of the cucumber *FRO* gene family, which showed that the responses of individual *CsFRO* genes to *A. brasilense* varied according to the plant nutritional status. Notably, under Fe sufficient conditions, only *CsFRO3*, but not *CsFRO1* or *CsFRO2*, responded to bacterial inoculation (Marastoni et al., 2019). These findings suggest that *A. brasilense* may differentially modulate components of the Fe-acquisition machinery depending on the Fe status of the host plant. Based on these observations, a transcriptomic analysis was undertaken in order to shed light on possibly alternative regulation mechanisms, and it was performed at 4 DAT, when *A. brasilense* inoculation enhanced FCR activity under both Fe sufficient and Fe deficient conditions.

Microarray-based transcriptomic analysis revealed that *A. brasilense* inoculation significantly modulated a comparable number of transcripts under both Fe deficient and Fe sufficient conditions. However, comparison of the differentially expressed transcripts showed that only 17 were shared between plants inoculated under Fe sufficient and Fe deficient conditions. Among these 17 transcripts, five were significantly up-regulated (log_2_FC> 1) and one was significantly down-regulated (log_2_FC< 1). The consistent regulation of these genes under both Fe conditions suggests that their expression is induced by *A. brasilense* inoculation independently of the plant’s Fe nutritional status. These genes are therefore likely associated with the plant response to microbe interaction. In fact, two of the up-regulated genes encode proteins belonging to the remorin family. Remorins (REMs) are plasma membrane associated proteins that are localised in membrane rafts, which function as key platforms for signal transduction processes involved in plant-microbe communication (Jarsch and Ott, 2011). In legumes, *REMs* expression has been linked with the successful establishment of the symbiosis with rhizobia (Lefebvre et al., 2010; Tóth et al., 2012), and, more in general, REMs have been shown to be involved in response to biotic and abiotic stress in different plant species and might be involved in abscisic acid-activated signalling pathway (Gouguet et al., 2021). In *Cucumis sativus* different REMs resulted to be modulated in presence of powdery mildew infection (Xu et al., 2017), further confirming their involvement in plant-microbe interaction. *Azospirillum* inoculation also induced the up-regulation of a gene encoding a Jackdaw (JKD) zinc finger protein, which is expressed in root cortical cells and participates in the signalling pathway controlling the differentiation of hair and non-hair root cells (Rongsawat et al., 2021). Interestingly, JKD is accounted as core regulator of how root tissues are patterned, mainly acting through the interaction with transcription factors Short-Root (SHR)/Scarecrow (SCR) (Welch et al., 2007). Recent reviews about the mechanisms used by PGPR to modulate root architecture suggest the involvement of SHR/SCR and PLETOHORA (PLT) networks as key endogenous regulators of the stem cell niche and meristem dynamics (Verbon and Liberman, 2016). In this scenario, the modulation of *JKD* expression by *A. brasilense* may indicate that this bacterium leverages this molecular pathway to drive the alterations in root architecture already documented in previous studies (Pii et al., 2016), in line with the established role of PGPR in shaping root development (Grover et al., 2021).

This observation was further corroborated by the up-regulation of a SKU5-like 3 protein induced by *A. brasilense* independently of the Fe nutritional status. Indeed, this gene encode a GPI-anchored family proteins which are essential for cell polar expansion and cell wall synthesis of roots in *Arabidopsis* (Zhou, 2019). Indeed, polarized expansion of the root cells contributes to the further growth of the root apparatus, allowing the plant to explore a higher volume of soil and to increase nutrient uptake, while maintaining mechanical strength and controlled organ shape (Goldy et al., 2025).

The only gene commonly down-regulated by *A. brasilense* inoculation in both Fe sufficient and Fe deficient cucumber plants encoded an AP endonuclease 2 (APEX2). This enzyme is involved in the base excision repair (BER) pathway, which repairs DNA lesions typically caused by reactive oxygen species (ROS), and plays a role in DNA strand break repair as well as in maintaining genomic integrity through active DNA demethylation (Li et al., 2023). The consistent downregulation of *APEX2* under both Fe conditions suggests that *A. brasilense* inoculation may influence the transcriptional regulation of DNA repair-related processes independently of the plant Fe nutritional status. Considering the role of *APEX2* in genome maintenance, its modulation may indicate an altered requirement for specific DNA repair pathways in response to the plant-bacterium interaction, a hypothesis that warrants further investigation. It should be noted that the differential-expression analysis was specifically designed to assess the transcriptional effect associated with *A. brasilense* inoculation within each Fe nutritional condition.

### 4.1 GO enrichment analysis

Gene Ontology (GO) enrichment analysis identified different sets of biological processes associated with *A. brasilense* inoculation within the two Fe nutritional regimes. Under Fe sufficient conditions, the bacterial treatment was associated with changes in a relatively restricted set of functional categories, whereas under Fe deficiency it affected a different and more diversified range of processes. These results are specifically interpreted here as transcriptional responses associated with bacterial inoculation within each nutritional context.

#### 4.1.1 *Azospirillum brasilense*-inoculated Fe sufficient cucumber

Under Fe sufficient conditions, bacterial inoculation led to the enrichment of broad BP terms, including *cellular process* and *metabolic process*. Within these two terms, it induced the significant down-regulation of several genes potentially involved in stress responses. These include bZIP transcription factors, which interact with stress-related proteins (Guo et al., 2024), cysteine-rich receptor-like protein kinases (Zhang et al., 2023) and α,α-trehalose-phosphate synthase a key enzyme in trehalose biosynthesis. Trehalose is a non-reducing sugar that contributes to abiotic stress protection by acting as an osmoprotectant (Hassan et al., 2023). Additionally, the previously discussed *APEX2* gene was also down-regulated. In contrast, among the up-regulated genes within the same BP terms, *A. brasilense* significantly modulated genes associated with plant growth and hormone signalling. In particular, the TFIID transcription factor, involved in RNA polymerase II recruitment (Lago et al., 2005) was up-regulated, potentially enhancing transcriptional capacity and supporting metabolic activity. A protein kinase and a DOF zinc finger protein, both implicated in hormone-mediated signalling pathways regulating plant growth, were also up-regulated (Laurie and Halford, 2001; Noguero et al., 2013). Moreover, two *ERFs* were up-regulated. Although *ERF*s are generally associated with stress responses, in the absence of stress they primarily regulate plant growth, development, and metabolic homeostasis (Yu et al., 2025). Overall, under Fe sufficient conditions, *A. brasilense* appears to shift the cucumber transcriptional profile by attenuating stress-response pathways while promoting growth- and development-related processes.

Only one significantly down-regulated gene was detected exclusively within the *cellular process* term and it encodes a zinc transporter. The reduced expression of this gene may indicate that, under Fe sufficient conditions, *A. brasilense* decreases zinc transporter activity, possibly because of the tight crosstalk between zinc and iron homeostasis (Xie et al., 2019). This transcriptional response may reflect adjustments in pathways associated with metal homeostasis, consistent with the known crosstalk between zinc and iron nutrition.

Regarding genes significantly modulated exclusively within the *metabolic process* term, *A. brasilense* appears to exert a consistent effect by down-regulating genes generally associated with stress responses. Among these, *β-glucosidase* was found to be down-regulated. This class of enzymes is involved in multiple metabolic processes, including chemical defence and phytohormone activation. In particular, specialized metabolites involved in plant defence are often stored in a glycosylated form to prevent toxicity and are subsequently activated through deglycosylation by β-glucosidases in response to stress, depending on substrate specificity (Stathaki et al., 2024). In addition, another down-regulated gene encodes a berberine bridge enzyme-like protein. Members of this family, particularly oligogalacturonide (OG) oxidases, are involved in the inactivation of OGs, whose production is typically induced during pathogens colonization or mechanical damage (Salvati et al., 2025). Similarly, the down-regulation of a Fe2OG dioxygenase domain-containing protein suggests a potential reduction in catalytic activity associated with a wide range of oxidative reactions. These enzymes are known to participate in secondary metabolism, hormone biosynthesis (including ethylene and gibberellins), and DNA repair processes (Farrow and Facchini, 2014). Their reduced expression may therefore indicate a lower activation of oxidative stress-related pathways. Finally, a gene encoding an ACC synthase, a key enzyme in ethylene biosynthesis, was also down-regulated (Morgan and Drew, 1997), suggesting a reduced transcriptional investment in pathways associated with ethylene biosynthesis. Taken together, the coordinated down-regulation of these genes is consistent with the hypothesis that *Azospirillum* attenuates stress- and defence-related metabolic pathways and ethylene biosynthesis, while promoting a shift toward processes associated with plant growth and development. Indeed, among the genes up-regulated following PGPR inoculation and belonging to *metabolic process*, two NADH-dependent nitrate reductases were identified, together with the already discussed SKU5-like 3 protein. The induction of nitrate reductases is consistent with a possible modulation of nitrogen assimilation-related processe, as these enzymes catalyse the rate-limiting step of nitrate reduction and are tightly linked to cellular redox balance and energy metabolism (Liu et al., 2026), which is often associated with increased plant growth. This observation is consistent with the known ability of PGPR to stimulate nitrogen metabolism and improve nutrient use efficiency (Kuan et al., 2016; Calvo et al., 2019; Lee et al., 2020). Interestingly, since Fe is an essential cofactor of nitrate reductase (Borlotti et al., 2012), the improved Fe nutritional status in *Azospirillum*-inoculated cucumber plants (Pii et al., 2016) is consistent with the observed upregulation of *nitrate reductase* transcript. In addition, a member of the ACT repeat-containing protein (ACR) family, specifically an ACR7-related protein, was up-regulated. ACR proteins have been proposed to act as amino-acid–sensing regulators related to glutamine metabolism and nitrogen status in plants (Hayakawa et al., 2006; Kudo et al., 2008; Sung et al., 2011; Takabayashi et al., 2016), suggesting that ACR7-related induction may contribute to the adjustment of amino acid pools and N signalling in response to *Azospirillum* inoculation (Zuluaga et al., 2026a).

Interestingly, the remaining BP terms significantly enriched by *A. brasilense* in Fe sufficient cucumber were associated with RNA-related processes, including RNA biosynthesis, transcription, and their regulation. The modulation of multiple transcription factors, such as bZIP, DOF, and ethylene-responsive proteins, may indicate that the bacterium affects host regulatory networks at the transcriptional level. This pattern is in line with transcriptomic studies showing that PGPR broadly reprogram host gene expression, including transcription factor families involved in hormone signalling, nutrient uptake and stress responses (Camilios-Neto et al., 2014; Spaepen et al., 2014; Samaras et al., 2022).

Focusing on the MF category, *A. brasilense* significantly enriched several *binding*-related terms, within which multiple genes were down-regulated. Among these, one encoded a HEC3 transcription factor which is known to regulate key developmental processes such as photomorphogenesis and cell behaviour at the shoot apical meristem (Gaillochet et al., 2018). The reduced expression of this transcription factor may reflect a reprogramming of developmental pathways toward a more energy efficient state under non-stress conditions. Indeed, *A. brasilense* did not induce any changes in the aboveground biomass of cucumber plants, independently of the Fe nutritional status (Pii et al., 2016). Additionally, PGPR inoculation induced the down-regulation of a calmodulin-binding protein. This class of proteins is involved in Ca^2+^-mediated signalling, a central component of plant responses to environmental stimuli, including both biotic and abiotic stresses (Virdi et al., 2015; Zeng et al., 2023). The downregulation of the calmodulin-binding protein suggests a reduced activation of calcium-dependent signalling cascades involved in responses to stress. Another down-regulated gene encoded an ABC transporter domain-containing protein. ABC transporters mediate the movement of a wide range of substrates, including secondary metabolites, phytohormones, and xenobiotics, and are often associated with detoxification processes and stress adaptation (Hwang et al., 2016).

Therefore, also the significantly modulated genes within the MF terms support the ability of *A. brasilense* to attenuate stress-related responses under Fe sufficiency conditions, thereby preserving energy and redirecting resources toward enhancing the plant’s metabolic and energetic capacity. This is further supported by the upregulation of a gene encoding an AAA+ ATPase domain-containing protein. AAA+ ATPases are highly conserved molecular machines that convert the energy derived from ATP hydrolysis into mechanical work, driving processes such as protein unfolding, complex disassembly, and proteolysis (Khan et al., 2022). Interestingly, in cucumber, the AAA ATPase CsARN6.1 is a major QTL for waterlogging tolerance. It promotes adventitious and lateral root formation, and its ATPase activity is essential for this effect (Xu et al., 2018). In this context, the upregulation of AAA+ ATPases in *Azospirillum*-treated roots could support a stronger or more plastic root systems, an improved tolerance to stresses that PGPR often mitigate (waterlogging, salt, oxidative stress) and the maintenance of protein and organelle homeostasis during PGPR-induced metabolic activation.

The last significantly enriched MF term was the *catalytic activity*, within which several genes were significantly modulated. Among the down-regulated genes, a SGNH hydrolase domain-containing protein was identified. Members of this family are involved in the modification of cell wall polysaccharides through acylation and deacylation reactions, contributing to the structural remodelling of plant cell walls, particularly under abiotic stress conditions (Hong et al., 2008; Anderson et al., 2022). The reduced expression of this gene may therefore suggest a decreased requirement for stress-related cell wall remodelling processes. Consistently, the down-regulation of a β-1,6-N-acetylglucosaminyltransferase family protein and a N-acetyltransferase further supports the hypothesis of a general suppression of stress-associated metabolic pathways. β-1,6-N-acetylglucosaminyltransferase play a key role in N-glycan biosynthesis, in particular under stress conditions (Yoo et al., 2021), whereas N-acetyltransferases are essential enzymes involved in the N-terminal proteins acetylation, which is a crucial modification for protein stability, plant development and stress tolerance (Pożoga et al., 2022).

In contrast, the up-regulation of a PAPS synthase highlights the activation of sulphur metabolism. This enzyme catalyses the conversion of inorganic sulphate into PAPS, the universal sulphur donor required for the biosynthesis of sulphated compounds, including phytohormones and secondary metabolites (Mueller and Shafqat, 2013).

#### 4.1.2 GO enrichment analysis in *A. brasilense*-inoculated Fe deficient cucumber

In Fe deficient cucumber plants, the inoculation with *A. brasilense* significantly enriched several BP terms. The first significantly enriched term was the *transmembrane transport*, within which three genes encoding a cationic amino acid transporter, a sulphate transporter and a sugar transporter were up-regulated. The up-regulation of a cationic amino acid transporter, which contribute to phloem loading and are expressed in vascular tissues (Dinkeloo et al., 2018), may reflect an increased demand for amino acid redistribution within the plant. Amino acids not only serve as building blocks for protein synthesis but also act as nitrogen carriers and precursors of signalling molecules, particularly under stress conditions (Heinemann and Hildebrandt, 2021).

Similarly, the induction of a sulphate transporter may indicate the involvement of sulphur acquisition-related responses. Notably, sulphur metabolism is closely linked to iron homeostasis, as in Fe deficiency is induced a significant increase in sulphur demand, thereby inducing sulphur uptake mechanisms (Astolfi et al., 2021). Additionally, sulphur is essential for the synthesis of key metabolites such as cysteine, methionine, glutathione which are particularly important under Fe deficiency (Astolfi et al., 2021). Enhanced sulphate uptake may therefore support both stress mitigation and metabolic adjustments induced by *A. brasilense*. In addition, the up-regulation of a sugar transporter may be consistent with transcriptional adjustments associated with carbohydrate transport and distribution. Sugar transporters play a central role in distributing carbohydrates from source to sink tissues and are often induced during plant-microbe interactions and under stress conditions, where they support metabolic adjustments and energy redistribution required for stress adaptation (Liu et al., 2025).

The enrichment of several BP terms related to RNA metabolism and transcriptional regulation in Fe deficient plants suggested that *A. brasilense* inoculation induced a substantial reprogramming of gene expression. In detail, within the *RNA biosynthetic process*, *transcription* and *transcription DNA-dependent* terms the up-regulation of an AP2/ERF domain-containing protein and a NAC-domain containing protein was observed. Members of the AP2/ERF family are key regulators of plant responses to abiotic stress and nutrient deficiency, including iron limitation, and play a central role in integrating hormonal and environmental signals (Yang et al., 2022; Ma et al., 2024). Similarly, NAC transcription factors are widely recognized as major regulators of stress responses and developmental processes, including root architecture, fruit ripening and adaptation to nutrient stress (Zheng et al., 2025). The up-regulation of these transcription factor families suggested that *A. brasilense* activated signalling pathways that enhance plant adaptive responses under Fe deficiency. In contrast, the down-regulation of a WRKY-related transcription factor, two bZIP transcription factors, and an ERF might suggest a suppression of specific stress and defence-related pathways. Indeed, as previously observed, ERF and bZIP are generally involved in the stress signalling. Similarly, also WRKY TF are key regulators of plant immunity and stress responses (Yang et al., 2025). Overall, these results indicated that *A. brasilense* may have induced a selective transcriptional reprogramming under Fe deficiency, most-likely promoting alternative adaptive and developmental pathways.

Moreover, within the *RNA metabolic process*, *A. brasilense* also induced the up-regulation of a gene encoding a lysyl-tRNA synthase, a key enzyme in protein translation that catalyse the attachment of lysine to its cognate tRNA (Wu et al., 2007). Interestingly, the expression of a lysyl-tRNA synthase has previously been reported in Fe deficient tomato roots, suggesting a potential role in stress adaptation and enhanced protein synthesis under nutrient limitation (Giritch et al., 1997).

Furthermore, *A. brasilense* inoculation significantly enriched the *cellular process*, *metabolic process*, as previously seen also in Fe sufficient conditions, and *biosynthetic process* terms. Within these terms, it up-regulated a cellulose synthase-like protein suggesting an activation of cell wall biosynthesis and remodelling. Indeed, cellulose synthase-like (CSL) proteins are involved in the synthesis of hemicelluloses and play a key role in cell wall expansion and root development (Mokshina, 2025). This observation is coherent with the enhanced root architecture observed in the Fe deficient cucumber inoculated with *A. brasilense* (Pii et al., 2016).

In contrast, several genes involved in primary and secondary metabolism were down-regulated, including a NADH-GOGAT, a riboflavin synthase, a 6,7-dimethyl-8-ribityllumazine synthase, a nicotianamine synthase, and a GMP synthase. NADH-GOGAT is a key enzyme in N assimilation, and previous studies have demonstrated that Fe deficiency enhanced the production of carbon skeletons, such as 2-oxoglutarate, required for amino acid biosynthesis, thereby sustaining NADH-GOGAT expression in cucumber roots despite reduced nitrate assimilation (Borlotti et al., 2012). In contrast, the down-regulation of NADH-GOGAT observed in this study might suggest that *A. brasilense* could alter nitrogen metabolism under Fe deficiency, potentially reducing the reliance on the GS/GOGAT cycle or improving nitrogen use efficiency, thus diminishing the need for its up-regulation.

Within Fe deficient plants, *A. brasilense* inoculation down-regulated genes encoding riboflavin synthase and 6,7-dimethyl-8-ribityllumazine synthase. Riboflavin biosynthesis and release have previously been associated with Fe mobilization in cucumber under Fe limitation (Bosch et al., 2024). Therefore, their downregulation shows that bacterial inoculation modulates riboflavin-related processes in plants already experiencing Fe deficiency, possibly altering the transcriptional investment in endogenous Fe-mobilization mechanisms. Similarly, inoculation down-regulated a gene encoding nicotianamine synthase. Since nicotianamine contributes to Fe transport and homeostasis (Li and Lan, 2017; Zha et al., 2022), this result might indicate that A. brasilense also affects components of internal Fe allocation within the Fe deficient nutritional context. Collectively, these transcriptional changes support an effect of bacterial inoculation on Fe-related processes beyond the canonical Strategy I components.

The simultaneous increase in FCR activity and downregulation of riboflavin- and nicotianamine-related transcripts should not necessarily be considered contradictory, since these responses represent distinct components of Fe acquisition and homeostasis. FCR activity reflects the capacity for Fe(III) reduction at the root surface, whereas riboflavin-related processes contribute to Fe mobilization and nicotianamine is primarily involved in internal metal chelation and distribution. These processes may therefore follow different temporal and regulatory dynamics. Moreover, cucumber FCR activity is associated with a gene family comprising at least three members. Marastoni et al. (2019) showed that, under Fe sufficient control conditions, *A. brasilense* increased FCR activity while *CsFRO1* and *CsFRO2* expression remained unchanged and *CsFRO3* was up-regulated. The same study also found that, under combined Fe and Cu deficiency, inoculation increased FCR activity without a corresponding induction of the three analysed *CsFRO* transcripts. Although these nutritional conditions are not identical to those used here, the findings demonstrate that the relationship between FCR activity and *CsFRO* transcript abundance can be isoform-, time- and nutrient-dependent. The pattern observed in the present study may therefore involve differential regulation of FRO isoforms or post-transcriptional mechanisms. However, since tissue Fe concentration and riboflavin or nicotianamine levels were not determined, it cannot be interpreted as evidence of partial recovery from Fe deficiency or reduced reliance on endogenous Fe-acquisition mechanisms.

Finally, the down-regulation of a GMP synthase, a key enzyme in purine nucleotide biosynthesis (Huang et al., 2022), in Fe-deficient plants inoculated with *A. brasilense* may be indicative of transcriptional adjustments affecting nucleotide biosynthesis and N-related metabolic processes towards a probable PGPR-enhanced Fe acquisition. This is consistent with the capacity of *A. brasilense* to reprogram host carbon and nutrient use specifically to support Fe uptake and translocation under stress (Pii et al., 2015, 2016; Marastoni et al., 2019; Housh et al., 2021).

Focusing on the gene shared between the *cellular process* and *metabolic process* terms, *A. brasilens*e down-regulated two genes encoding enzymes involved in the BER pathway, N-glycosylase/DNA lyase and *APEX2*, a genome defence mechanism to repair DNA damage caused by endogenous and exogenous factors (Roldán-Arjona et al., 2019). Given the role of these enzymes in DNA repair pathways often associated with oxidative lesions, their concurrent downregulation suggests that *A. brasilense* may affect the transcriptional regulation of genome maintenance processes.

In contrast, the up-regulation of glutamate decarboxylase (*GAD*) points to a transcriptional modulation potentially involving GABA shunt, a metabolic pathway tightly linked to plant stress acclimation (Wu et al., 2020; Ansari et al., 2021). Increased GABA biosynthesis via GAD is a common response to abiotic stress and its accumulation enhances tolerance by supporting antioxidant defence, ion homeostasis and TCA-cycle associated energy supply (Ji et al., 2020; Wu et al., 2020; Ansari et al., 2021; Wang et al., 2023; Akter et al., 2024). Notably, in cucumber, GABA application alleviates Fe-deficiency symptoms by stimulating Fe-uptake mechanisms through auxin-dependent signalling (Guo et al., 2020). Thus, *GAD* up-regulation in *A. brasilense*-inoculated plants may reflect activation of GABA-mediated adaptive responses that bolster resilience under Fe deficiency. In addition to the metabolic and signalling adjustments mediated by the GAD-GABA pathway, *A. brasilense* also appeared to affect genes potentially related to membrane composition. In fact, the up-regulation of a sterol-3β-glucosyltransferase indicated that *A. brasilense* promoted the modulation of membrane lipid composition. This enzyme catalyses the glycosylation of sterols, contributing to membrane stability and fluidity, which are essential for maintaining cellular integrity under stress conditions (DeBolt et al., 2009; Rogowska and Szakiel, 2020).

Furthermore, among the significantly modulated genes belonging exclusively to *metabolic process* term, *A. brasilense* enhanced the expression of several genes involved in stress response, secondary metabolism, redox regulation, and plant-microbe interactions, suggesting a coordinated metabolic reprogramming. Cytochrome P450 enzymes have different role in plants including regulation of plant hormone metabolism, oxidative reactions, and the biosynthesis of secondary metabolites, including compounds with defensive or signalling roles that may contribute to plant adaptation under nutrient stress conditions (Minerdi et al., 2023). Similarly, the up-regulation of an L-ascorbate oxidase suggested that *A. brasilense* may have influenced the cellular redox state. This enzyme, localized in the apoplast, catalyses the oxidation of ascorbic acid and plays a key role in regulating the redox balance of the apoplastic ascorbate pool (Venkatesh and Park, 2014). Interestingly, the induction of a Nod factor hydrolase points to the activation of mechanisms involved in the modulation of lipochitooligosaccharide signalling, which is central to plant-microbe interactions (Liang et al., 2014). Although typically associated with symbiotic relationships, the regulation of Nod factor-related enzymes suggests that *A. brasilense* may influence signalling pathways that facilitate beneficial colonization.

Furthermore, the up-regulation of a SKU5-like protein, as also previously observed in Fe sufficient conditions, supports the idea that *A. brasilense* promotes root development and cell expansion. Finally the up-regulation of an acyl-activating enzymes, the butyrate-CoA ligase, which catalyses the ATP-dependent activation of medium-chain fatty acids into CoA thioesters (Shockey et al., 2003), may suggest an effect of *A. brasilense* even on fatty acids metabolism under stress conditions.

Concerning the transport and localization BP terms, *A. brasilense* significantly modulated several genes within these terms. In particular, the bacterium induced the up-regulation of a gene encoding a peptide transporter, a membrane protein that mediates uptake and redistribution of small peptides and other organic nitrogen compounds (Rentsch et al., 2007; Zhang et al., 2020; Kanstrup and Nour-Eldin, 2022; Talevi and Bellera, 2022). In parallel, the observed down-regulation of NADH-dependent glutamate synthase, a key enzyme for root ammonium assimilation (Konishi et al., 2014), suggests that under Fe deficient conditions *A. brasilense* may favour coordinated transcriptional changes involving ammonium assimilation and organic N transport. Interestingly, the PGPR inoculation also promoted the expression of two copper (Cu) transport protein family members. Iron and Cu metabolisms are closely interconnected, as Strategy I plants reduce both metals through FRO-family metalloreductases whose activity is regulated by Fe and Cu availability (Puig et al., 2007; Jain et al., 2014; Marastoni et al., 2019; Guan et al., 2024). *A. brasilense* has previously been shown enhance root physiological activities in Fe deficient cucumber (Pii et al., 2016), which may also lead to an increased bioavailability of other mineral cations, including Cu (Pii et al., 2015; Brunetto et al., 2016). The up-regulation of Cu transporters in inoculated plants therefore suggests that *A. brasilense* not only improves micronutrient availability but also contributes to a coordinated adjustment of metal homeostasis under Fe deficiency. In contrast, the down-regulation of an aquaporin PIP, a plasma membrane protein that facilitates the transport of water and small molecules (Zhu et al., 2019), suggested a modification of water transport and membrane permeability. Aquaporins are tightly regulated under stress conditions, for instance, some PIP isoforms have been down-regulated under salinity stress to limit water loss (Zhu et al., 2019). Therefore, their reduced expression in this context may reflect an *A. brasilense*-induced adjustment of root water transport and membrane permeability, potentially contributing to adjustments in water transport and membrane permeability under Fe deficient conditions.

Additionally, the down-regulation of an equilibrative nucleoside transporter point to a reprogramming of nucleotide metabolism and transport. Equilibrative nucleoside transporters in plants play a key role in the membrane transport of purine and pyrimidine nucleosides for nucleic acid synthesis (Girke et al., 2014, 2015), and their reduced expression may indicate altered nucleotide turnover or a shift in metabolic priorities under these conditions.

The last BP terms significantly enriched under Fe deficient conditions, *response to stimulus* and *response to stress*, were also enriched under Fe sufficient conditions. Interestingly, the response of calmodulin-related genes to *A. brasilense* differed according to the plant Fe nutritional status. While two calmodulin-binding protein genes were up-regulated under Fe deficient conditions, a distinct calmodulin-binding protein gene was down-regulated under Fe sufficiency. Although the genes involved were not the same, these observations indicate that components of Ca^2+^-related signalling networks are responsive to bacterial inoculation under both nutritional conditions. This context-dependent modulation is consistent with the ability of PGPR to fine-tune stress signalling, dampening responses under optimal conditions and enhancing adaptive pathways under nutrient limitation. Consistently, among the MF terms associated with binding-related functions, *A. brasilense* also induced the up-regulation of an EF-hand protein, that corroborates the activation of Ca^2+^-dependent signalling pathways in inoculate plants. In fact, the majority of the Ca^2+^-binding or transporter proteins in plants, including Calmodulin proteins, possess EF-hand domains (Kaur et al., 2022).

Within the same MF term, *A. brasilense* inoculation induced the down-regulation of an E3 that points to a modulation of protein turnover *via* the ubiquitin-proteasome system (UPS) (Wang et al., 2022). E3 ligases are key determinants of substrate specificity in protein degradation and are often involved in the regulation of stress-responsive pathways (Wang et al., 2022). Their reduced expression may indicate an *A. brasilense*-induced adjustment of UPS-mediated protein turnover in stress-related pathways, potentially contributing to stabilized cellular homeostasis under Fe deficient conditions.

Additionally, the down-regulation of an alanine-glyoxylate aminotransferase, an enzyme involved in amino acid interconversion and photorespiratory metabolism (Liepman et al., 2019), may further support a reprogramming of N and C metabolism. In line with the observed down-regulation of NADH-GOGAT and the shift toward organic N uptake, its reduced expression induced by *A. brasilense* may reflect a transcriptional reorganization of genes related to C and N metabolism aimed at optimizing resource allocation under Fe deficiency.

An important limitation of the present study is that, beyond root FCR activity, complementary physiological, biochemical and microbiological parameters were not measured concurrently with the transcriptomic analysis. Previous studies using the same cucumber - A*. brasilense* biological system showed that inoculation increased leaf Fe concentration, chlorophyll content and biomass under Fe-limiting conditions in calcareous soil (Pii et al., 2015), and enhanced rhizosphere acidification, root development and ^57^Fe uptake under hydroponic conditions (Pii et al., 2016). Furthermore, Marastoni et al. (2019) reported inoculation-dependent changes in root architecture, FCR activity and root Fe concentration and showed that the cucumber FRO family comprises at least three genes displaying distinct responses to the nutritional regime and bacterial inoculation. These findings provide a physiological and molecular framework for the transcriptional responses observed here but do not directly demonstrate corresponding changes in Fe accumulation, metabolite production or bacterial colonisation in the present experiment. Therefore, the functional interpretations proposed should be regarded as working hypotheses requiring validation through integrated measurements of plant Fe status, root exudation, rhizosphere chemistry and bacterial activity.

## 5 Conclusions

**Figure 5:**
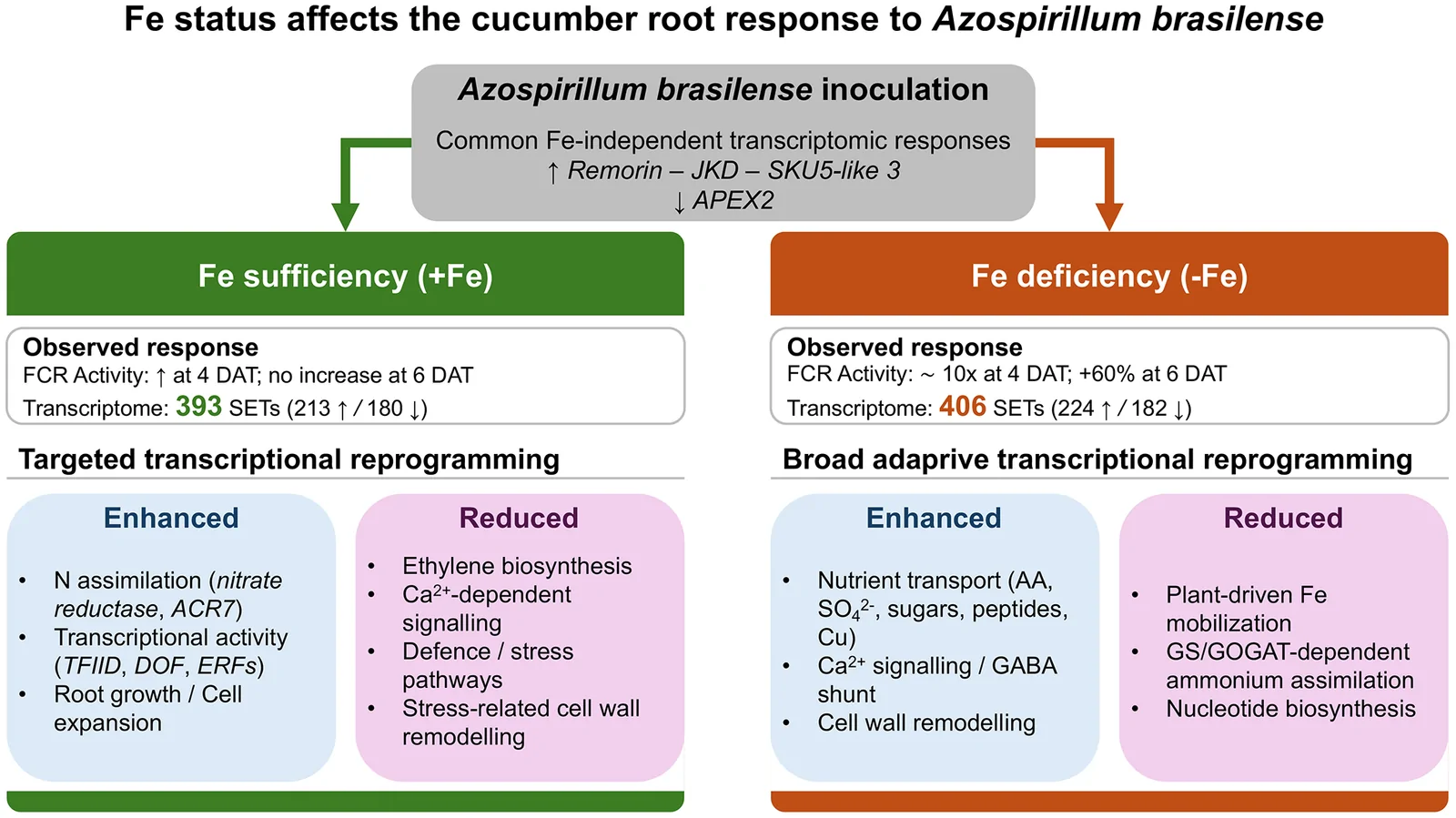
Schematic summary of the main responses associated with *Azospirillum brasilense* inoculation in Fe sufficient and Fe deficient cucumber plants. The figure reports the experimentally observed increase in root FCR activity and the principal functional categories associated with the differentially expressed transcripts identified separately under the two Fe nutritional regimes. The transcriptional categories shown should not be interpreted as evidence of corresponding changes in metabolic fluxes, metabolite concentrations or whole-plant physiological performance.

Taken together, these results indicate that *A. brasilense* increased root FCR activity at 4 DAT and induced predominantly distinct transcriptional responses in Fe sufficient and Fe deficient cucumber plants (**Figure 5**). These findings show that bacterial inoculation was associated with predominantly distinct transcriptional responses when analysed separately under Fe sufficient and Fe deficient conditions, supporting the hypothesis that plant Fe nutritional status may shape the response to *A. brasilense*.

Under Fe sufficient conditions, the bacterium exerts a relatively targeted effect, primarily related to the downregulation of several stress- and defence-associated transcripts and the modulation of genes related to transcriptional regulation and N metabolism. This transcriptional profile is consistent with a possible attenuation of stress-associated signalling and modulation of growth- and metabolism-related processes.

In contrast, under Fe deficiency, *A. brasilense* inoculation was associated with a broader range of transcriptional changes involving transport systems for amino acids, sulphate, sugars, peptides and metals, together with transcriptional regulation, stress-related signalling and C and N metabolism. The modulation of genes related to riboflavin biosynthesis, nicotianamine production and nutrient transport suggests that bacterial inoculation affects processes connected with Fe mobilization and homeostasis in plants experiencing Fe deficiency. The limited overlap between the responses detected under the two Fe regimes indicates that the transcriptional changes associated with inoculation were predominantly distinct in Fe sufficient and Fe deficient plants. Although tissue Fe concentration, chlorophyll status, biomass, oxidative stress markers and nutrient-use efficiency were not assessed concurrently with transcriptomic profiling in the present experiment, previous studies using the same cucumber - A*. brasilense* biological system have already demonstrated enhanced Fe uptake and accumulation, recovery of chlorophyll levels, increased biomass, rhizosphere acidification and changes in root development under Fe-limiting conditions, including cultivation in calcareous soil. Therefore, the present results should not be regarded as stand-alone evidence of these physiological outcomes, but rather as molecular evidence that complements and helps interpret the previously reported phenotypic responses. Further studies integrating transcriptomic profiling with concurrent physiological and biochemical measurements will be useful to establish direct relationships between specific transcriptional signatures and plant Fe acquisition, stress responses and nutrient metabolism, as well as to assess the robustness of these responses under field conditions.

## Data Availability

The datasets presented in this study can be found in online repositories. The names of the repository/repositories and accession number(s) can be found below: https://www.ncbi.nlm.nih.gov/geo/, GSE331093.

## Supplementary material

Supplementary Figure 1 Schematic drawing representing the experimental plant, specifically reporting the treatments applied, the sampling times and the analyses carried out at each sampling time.

Supplementary Figure 2 Principal Component Analysis (PCA) of normalized transcriptomic data from cucumber roots grown under Fe sufficient and Fe deficient conditions, either non-inoculated or inoculated. The PCA was performed using the 2,000 transcripts showing the highest variance across all samples. Each point represents one biological replicate.

Supplementary Table 1 Sequences of forward and reverse primers used in RT-qPCR experiments.

Supplementary Table 2 Differential expression analysis of four selected transcripts identified by microarray analysis and validated by RT-qPCR. Corresponding log_2_ fold change (log_2_FC) values obtained from microarray. RT-qPCR data are reported as means ± SE of the relative amount of transcript.

Supplementary Table 3 Significantly modulated Gene Ontology (GO) terms in the comparison +Fe + *A. brasilense* vs +Fe. For each GO term, the associated genes and their corresponding log_2_ fold change (log_2_FC) values are reported.

Supplementary Table 4 Significantly modulated Gene Ontology (GO) terms in the comparison -Fe + *A. brasilense* vs -Fe. For each GO term, the associated genes and their corresponding log_2_ fold change (log_2_FC) values are reported.
